# Adapting the Bayley Scales of infant and toddler development in Ethiopia: evaluation of reliability and validity

**DOI:** 10.1111/cch.12371

**Published:** 2016-07-06

**Authors:** C. Hanlon, G. Medhin, B. Worku, M. Tomlinson, A. Alem, M. Dewey, M. Prince

**Affiliations:** ^1^ Department of Psychiatry, College of Health Sciences, School of MedicineAddis Ababa UniversityAddis AbabaEthiopia; ^2^ Institute of Psychiatry, Psychology and Neuroscience, Centre for Global Mental HealthKing's College LondonLondonUK; ^3^ Aklilu‐Lemma Institute of PathobiologyAddis Ababa UniversityAddis AbabaEthiopia; ^4^ Department of Paediatrics and Child Health, College of Health Sciences, School of MedicineAddis Ababa UniversityAddis AbabaEthiopia; ^5^ Department of PsychologyStellenbosch UniversityStellenboschSouth Africa; ^6^ Health Services and Population Research Department, Institute of PsychiatryKing's College LondonLondonUK

**Keywords:** child development, Ethiopia, measurement, sub‐Saharan Africa, validation

## Abstract

**Background:**

There is a need for valid and reliable observational measures of early child development in low‐income and middle‐income country settings.

**Methods:**

The aims of the study were to adapt the Bayley Scales of Infant Development (Bayley III) for a rural Ethiopian setting and evaluate reliability and validity. The study was carried out between January 2008 and January 2009 in the Butajira demographic surveillance site, south central Ethiopia. The Bayley III was adapted to be socioculturally appropriate for a rural Ethiopian context. Nurses and high school graduates were trained in administration of the measure for 10 days. Inter‐rater reliability was evaluated (*n* = 60). Content, construct and convergent validity was then examined on a population‐based cohort of children at the ages of 30 (*n* = 440) and 42 months (*n* = 456). Mokken scale analysis was used to determine the scalability of items in unidimensional, hierarchical sub‐scales. The mean score was compared by age of child and by stunting status (less than −2 *z* scores below the standard height‐for‐age).

**Results:**

The intra‐class correlations between raters were above 0.90 for all sub‐scales of the child development measure. Some scale items were not contextually relevant and showed poor scalability. However, the majority of items scaled onto the existing sub‐scales of the international measure to form adequate‐to‐strong hierarchical scales with good internal consistency (Cronbach's α above 0.70 except for gross motor and expressive language sub‐scales). Item‐scale coefficients were good. The mean score of all sub‐scales was significantly higher in the older group of children (33.02 higher total score; *P* < 0.001) and in the children who were stunted (total Bayley score 2.58 (95% confidence interval 0.07 to 5.10) points lower at 30 months and 3.87 (1.94 to 5.81) points lower at 42 months.

**Conclusions:**

An adapted version of an international, observational measure of child development was found to be reliable, valid and feasible in a rural Ethiopian setting.

## Introduction

The developmental potential of an estimated 200 million children living in low‐income and middle‐income countries (LMICs) is not being realized, leading to adverse impacts upon educational attainment, adult earning capacity and ability to parent the next generation of children (Grantham‐McGregor *et al*. 2007). Contextually appropriate measurement of child development in LMICs is vital for research to quantify the extent of the problem, to compare across populations and settings and to target and evaluate interventions. Measures for detection and monitoring of individual‐level child developmental abnormalities in community or clinical settings are also needed. However, in both research and clinical settings in LMICs, the challenges to measurement of child development are numerous.

The construct of child development is complex, encompassing cognitive, sensorimotor and social‐emotional domains (Walker *et al*. 2007). Furthermore, the validity of child development measures that have been developed in Western, high‐income countries may not translate to LMICs, particularly in rural areas, because of the differing sociocultural context and lower levels of formal education (Sternberg *et al*. 2001). Feasibility is also a major concern, with respect to the availability of time, equipment and personnel with appropriate expertise to administer assessments. In rural healthcare settings in sub‐Saharan Africa, new measures of developmental delay in preschool children have been developed (Abubakar *et al*. 2010; Gladstone *et al*. 2010b). This has been carried out by undertaking careful qualitative work to identify culturally relevant items, reliability testing and piloting to inform item selection, establishment of population norms and evaluation of construct and concurrent validity (Abubakar *et al*. 2010; Gladstone *et al*. 2010b). The limitation of such an ‘emic’ approach (developing a measure from within a cultural setting) is that the applicability of the resulting scales to other LMIC settings is not known. In addition, the contextual specificity limits comparability across settings, and the lengthy process required to develop a psychometrically robust new measure is not possible in many settings.

The alternative ‘etic’ approach would be to adapt existing measures of child development from outside the culture, most often developed in high‐income, Western countries, and examine the applicability to the new setting. In Ethiopia, there are no ‘emic’ measures of early child development. However, previous research studies have used adapted forms of the Bayley Scales of Infant and Toddler Development (the Bayley Scales) (Aboud and Alemu 1995; Drewett *et al*. 2001; Bayley 2006). In those studies, highly educated university graduates were used to administer the scale, with limited generalizability to real‐world settings, and no formal assessments of reliability or validity of the Bayley Scales were reported.

The objectives of this study were to adapt the Bayley Scales to be socioculturally appropriate for a rural Ethiopian setting and to evaluate the reliability and content, construct and convergent validity of the adapted scale when administered by high school graduates.

## Methods

This study was carried out as part of the child outcomes and maternal mental disorders in Ethiopia (C‐MaMiE) study.

### Setting

The location of the study was Butajira, a predominantly rural area of Ethiopia, located about 130 km south of the capital city, Addis Ababa, in the Southern Nations Nationalities and Peoples Region. The validation study was conducted within the Butajira demographic surveillance site (Berhane *et al*. 1999), a field laboratory established nearly 25 years ago, and the reliability study recruited children from neighbouring sub‐districts. The characteristics of the women and children participating in the C‐MaMiE cohort have been described previously (Hanlon *et al*. 2009a). The majority of women live in rural areas and are non‐literate. Similar to other rural areas in Ethiopia, there is a high level of childhood stunting and infant morbidity from infectious disease (Medhin *et al*. 2010).

### Adaptation of the Bayley Scales

We used the Bayley Scales of Infant and Toddler Development, version III (Bayley 2006). The Bayley Scales assess developmental functioning and delay in children from 1 to 42 months of age across the following domains: cognitive, expressive and receptive language, and fine and gross motor. The Bayley Scale assessment is carried out by the professional assessor with input from the parent.

#### Translation

The instruction manual was translated into Amharic, the official language of Ethiopia, by a bilingual Ethiopian doctor who had worked for many years in the psychiatric outpatient clinic at Butajira hospital. The translated version was circulated to the project collaborators, including an Ethiopian paediatrician (B. W.) and Ethiopian child psychiatrist (Y. B.), and comments were obtained. Further minor modifications were made during initial piloting of the manual, under supervision of the paediatrician (B. W.).

#### Modification of test materials

1. Picture book

The Bayley Scales include a ‘picture book’, which has photographs of objects and people carrying out actions. As the photographs mostly depicted objects familiar in Western settings, for example, a tricycle, scissors and an aeroplane, a version more relevant for a rural Ethiopian setting was developed, including photographs of a chicken, a three‐legged stool and a cart. Furthermore, all photographs of people were of Ethiopians.

Stimulus book

Similarly, an adapted version of the Bayley ‘stimulus book’ was developed by an Ethiopian artist. The drawings of objects and actions were all modified to be relevant to a predominantly rural Ethiopian setting and to depict Ethiopian people.

Modification of Bayley items

Following the example of previous Ethiopia studies using the Bayley Scales (Aboud and Alemu 1995), we dropped the limits on timed items (cognitive sub‐scale items 61, 62, 63, 66, 70 and 82; fine motor sub‐scale items 62, 63, 64 and 65; gross motor sub‐scale items 69 and 70). Seven items from the gross motor scale were removed (items 47, 49, 54, 57, 58, 64 and 67) as they involved the use of stairs. Participants' homes were single‐storey dwellings without steps and are located several kilometres from the road, meaning that it was both impractical and culturally inappropriate to use pre‐made stairs in the assessment.

### Inter‐rater reliability of the Bayley Scales

#### Training

Training in administration of the adapted Bayley Scales was conducted over a total of 10 non‐consecutive days. The trainees included six general diploma level nurses who were working either in the local health centres or as outreach nurses in the district health office. The remaining nine trainees were data collectors on the existing C‐MaMiE project. All of the C‐MaMiE data collectors had completed secondary school education; two were enrolled in part‐time nursing training and three in part‐time teacher training. Five of the participants had previous experience administering the Bayley Scales to 12‐month‐old infants in another project.

During the first 2 days of training, the trainees were given theoretical background to the scale, taught how to optimize the setting for administration and introduced to each of the Bayley items in turn. The participants also watched the Bayley Scales training video. From day 3 onwards, to complement the ongoing theoretical training, trainees practised administration of the Bayley items on 2‐ and 3‐year‐old children. Training was led by the project co‐investigator (G. M.), who has a Masters in Applied Statistics and experience working with the Bayley Scale in Butajira, supported by an Ethiopian Consultant Paediatrician (B. W.) and an Ethiopian psychiatrist (A. A.). The Paediatrician took a prominent role in observing administration of the complete Bayley Scales by trainees, giving feedback and discussing the findings in detail with the trainees.

#### Reliability testing

After completion of the training, a practice reliability exercise was conducted. The trainees were divided up into groups of three or four. While one person administered the Bayley, all present (the test administrator and the observers) rated each of the items independently. This exercise was repeated so that each trainee had an opportunity to administer the Bayley. Any discrepancies in rating were discussed at length, and any misunderstandings were clarified.

At this stage, the project co‐ordinator was confident in the abilities of the trainees, and a formal evaluation of inter‐rater reliability was carried out. The trainees were grouped into five groups of three, each group combining nurses and C‐MaMiE data collectors. For each test, one person administered the full Bayley Scales, without interference from the observers, and all present rated the Bayley items independently and simultaneously. Each group member had an opportunity to administer the Bayley Scales on four occasions. The children were from a convenience sample of children aged between 2 and 3 years of age and living in rural sub‐districts around Butajira. The Bayley Scale assessment was carried out in the family home with the mother present.

Reliability was evaluated by calculating the intra‐class correlation coefficient for the total raw scores on each Bayley sub‐scale for the administrators and observers.

### Validation study

The content, construct and convergent validity of the Bayley Scales were assessed. These aspects of instrument validity are recognized as necessary to establish the cultural validity of a measure that has been developed in one cultural setting and is now being applied in a distinct context. To establish content validity, it is necessary to demonstrate that ‘the content of each item of the instrument is relevant to the phenomena in each culture being studied’ (Flaherty *et al*. 1988). Construct validity means ‘the extent to which the construct that the measure seeks to address is a real and coherent entity’ (Prince 2003). Convergent validity is indicated when a measure is associated with factors that are known to be associated with the construct that the instrument purports to measure (Prince 2003).

**Figure 1:**
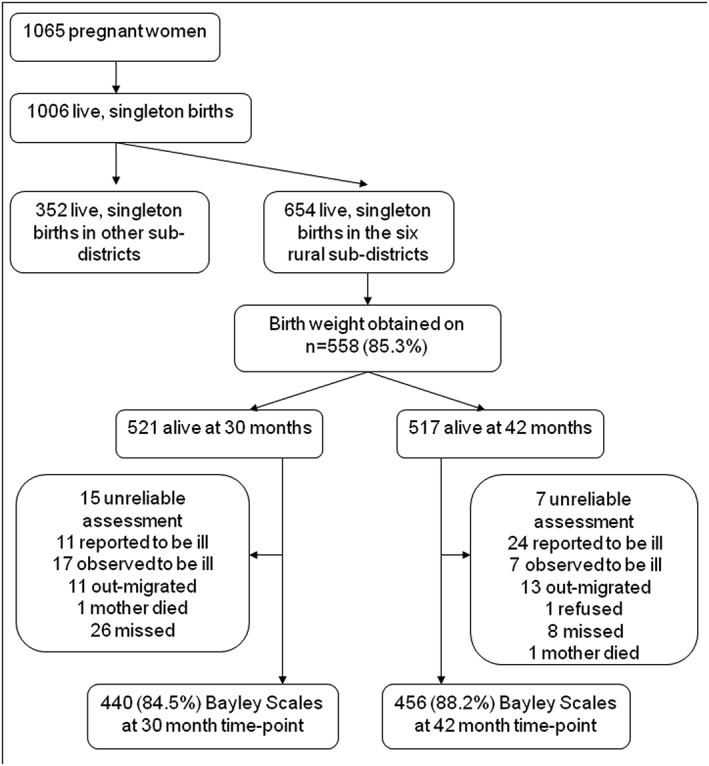
Flowchart for Bayley Scale assessments at 30 and 42 months.

The study participants for the validation study were part of the ongoing C‐MaMiE study. As has been described elsewhere in detail (Hanlon *et al*. 2009a), a population‐based sample of 1065 women in the third trimester of pregnancy was recruited from the Butajira demographic surveillance site (Berhane *et al*. 1999) between July 2005 and February 2006. The women, together with the children they gave birth to, are continuing to be followed up on a regular basis. Assessments using the adapted Bayley Scales were carried out on a representative subsample of the C‐MaMiE cohort children, selected because their birthweight had been measured within 48 h of birth (79.9% of eligible babies) (Fig. 1). As reported previously (Hanlon *et al*. 2009b), eligible women for whom birthweight was not obtained were more likely to be non‐literate and nulliparous but did not differ in terms of age, socio‐economic status, substance use or self‐reported health status.

Assessments were conducted when the children were 30 and 42 months of age. As for the reliability test, Bayley Scale assessments were carried out in the familiar environment of the child's home, with the mother present. The duration of assessments ranged from 30 to 60 min. Testing was deferred, or the child was excluded, if the child appeared to be ill or if the mother reported that the child was ill. Children were given a snack of some bread prior to testing.

### Content validity

The percentage of children passing a given Bayley Scale item was plotted to see if there was any evidence of content invalidity, which would be indicated by a low percentage of children passing the age‐appropriate item taking into account the level of difficulty.

### Construct validity: Mokken scale analysis

The items in the Bayley Scales are arranged in order of difficulty. We would, therefore, expect the items on the sub‐scales to be ordered in a hierarchical manner. To examine whether this hierarchical property was still present when the Bayley Scales were used in rural Ethiopia, we employed Mokken analysis (Mokken 1971). Mokken analysis utilizes non‐parametric item response theory to evaluate the presence of a hierarchical scale within a set of responses (Watson *et al*. 2008). In Mokken scales, the relative ordering of items is assumed to reflect ordering along an underlying latent trait. For each sub‐scale of the Bayley, the start point for inclusion of items in the Mokken analysis was the recommended start point for the age of the child. All subsequent items were included up to the last item, which was informative for the Mokken analysis. Item‐scale Loevinger coefficients for Bayley sub‐scale items were inspected, and items scoring <0.30 were excluded. The conditions of monotone homogeneity and double monotonicity were assessed, and items leading to violations were removed. The Loevinger H for each final scale was evaluated. A scale is considered dimensionally weak for Loevinger coefficients between 0.30 and 0.39, moderate for coefficients between 0.40 and 0.49 and strong for coefficients of 0.50 or higher (Mokken 1971). Internal consistency of the resulting Bayley sub‐scales was measured using Cronbach's α (Cronbach 1951).

### Convergent validity: association with expected predictors

As the Bayley Scales are a measure of child development, the mean raw score (uncorrected for age) for children at 30 months of age would be expected to be significantly lower than the mean raw score at 42 months of age. This was evaluated by comparing the mean scores at the two time points and using matched *t*‐test to evaluate statistical significance. Child undernutrition, particularly low height‐for‐age (stunting), is also known to be a robust predictor of child cognitive development (Grantham‐McGregor *et al*. 2007). Child anthropometric measures (height and weight) were carried out according to recommended procedures (WHO Expert Committee 1995) by the project data collectors and standardized to generate *z* scores using the 2006 WHO reference population (Monika *et al*. 2005). Children with *z* scores ≤ −2 were categorized as stunted. The mean Bayley sub‐scale and total scale scores in stunted vs. non‐stunted children were evaluated using an independent samples *t*‐test.

### Ethical considerations

Ethical approval for the study was obtained from Research Ethics Committees of the Ethiopian Science and Technology Agency and King's College London, UK. Healthcare costs of mothers and children participating in the C‐MaMiE cohort study were covered by the project throughout the study period.

## Results

### Inter‐rater reliability

A total of 60 assessments using the adapted Bayley Scales were conducted on 60 children. See Table 1. Consistently, high intra‐class correlation coefficients (>0.90) were found across the Bayley sub‐scale scores indicating excellent inter‐rater reliability (Cicchetti 1994). There was no evidence of a difference in reliability of administration by nurses compared with C‐MaMiE data collectors.

**Table 1:** Intra‐class correlation for each sub‐scale (*n* = 60)

Bayley sub‐scale	Intra‐class coefficient (95% confidence interval)
Cognitive	0.91 (0.87, 0.95)
Receptive language	0.95 (0.93, 0.97)
Expressive language	0.99 (0.98, 0.99)
Fine motor	0.95 (0.93, 0.97)
Gross motor	0.96 (0.95, 0.98)

### Content validity

**Figure 2:**
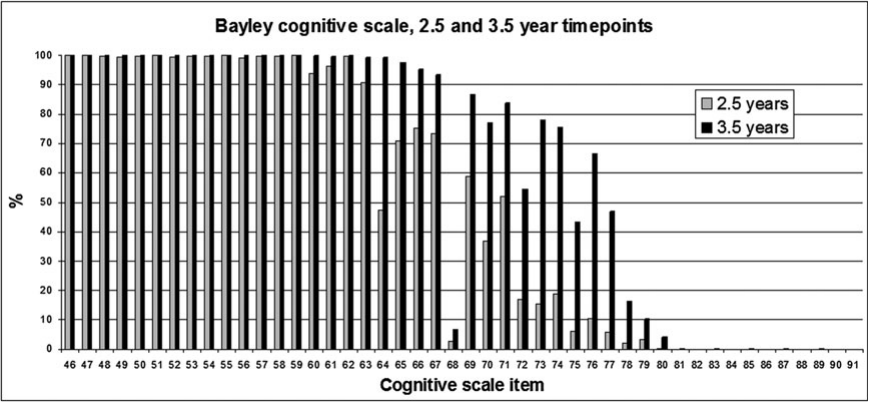
Percentage of children passing items on the Bayley cognitive sub‐scale.

Some items were passed by a very low percentage of children when compared with neighbouring items in the Bayley scale which would be expected to be of a similar level of difficulty (Supporting Information). At both time points, very few children passed item 68 on the cognitive scale (matching three colours) or items 47 (snips paper), item 51 (cuts paper) and item 55 (cuts on line) on the fine motor scale. Some items were problematic at the 42 month time point only: items 35 (identifies colours) and 38 (understands his/her pronouns) in the receptive language sub‐scale and items 38 (uses plurals) and 41 (names four colours) in the expressive language sub‐scale. The item frequencies for the cognitive sub‐scale are illustrated graphically in Fig. 2.

### Construct validity: Mokken scale analysis

See Table 2. For each of the Bayley sub‐scales, the majority of items formed a hierarchical scale at both age time points, with the number of included items ranging from 16 to 21, except for expressive language (*n* = 13) and gross motor (*n* = 7) at the 42‐month time point. Internal consistency, indicated by Cronbach's α, was high for all except the gross motor scale at the 42‐month time point. In most cases, the Loevinger H coefficient indicated a ‘strong’ scale (>0.50).

**Table 2:** Hierarchical scale and internal consistency properties of the Bayley sub‐scales

	Cognitive		Language receptive		Language expressive		Fine motor		Gross motor	
Time point (months)	30	42	30	42	30	42	30	42	30	42
Sample size	440	456	440	456	440	456	440	456	440	456
Start point for scale	Item 60	Item 67	Item 22	Item 28	Item 23	Item 30	Item 37	Item 43	Item 51	Item 59†
End point for scale	Item 80	Item 91	Item 42	Item 49	Item 46	Item 48	Item 63	Item 66	Item 72	Item 72
Number of items in final Mokken scale	16	16	17	18	21	13	20	16	18	7
Number of items not fitting into Mokken scale	5	9	4	4	3	6	9	8	1	5
Loevinger H scale coefficient	0.56	0.45	0.51	0.69	0.63	0.51	0.61	0.47	0.58	0.49
Cronbach's α	0.77	0.74	0.73	0.82	0.82	0.69	0.79	0.71	0.80	0.60
Excluded items	64, 65, 67, 68, 69	68, 76, 81, 82, 84, 86, 88, 90, 91	22, 35, 38, 40	33, 34, 35, 49	28, 41, 45	32, 33, 35, 37, 38, 41	35, 36, 40, 41, 43, 46, 51, 55, 62	47, 48, 49, 51, 55, 64, 65, 66	56	62, 63, 66, 69, 70

† The start point for 42‐month‐old children was item 57, but this item and 58 were not administered because they required steps.

Within the cognitive sub‐scale, the item 68 (matches three colours) did not scale in the Mokken analysis for either time point. The following items did not scale at the 30‐month time point but did scale at the 42‐month time point: item 64 (matches pictures), item 65 (representational play), item 67 (imitates a two‐step action) and item 69 (imaginary play). The following items did not scale at the 42‐month time point: item 76 (discriminates pictures), item 81 (identifies three incomplete pictures), item 82 [object assembly (dog)], item 84 (spatial memory), item 86 (number constancy) and item 88 (classifies objects). Almost no children passed items 81 to 86.

For the receptive language scale, item 35 (identifies colours) did not scale at either time point. The following items did not scale at the 30‐month time point but did scale in the 42‐month time point: item 22 (identifies three clothing items), item 38 [understands pronouns (his, her)] and item 40 (understands more). Item 22, the start point for 30‐month‐old children was passed by nearly all children (98.9%), and no children passed items 38 or 40. The following items did not scale at the 42‐month time point: item 33 (understands possessives) and item 34 (understands verbs ending in ‘ing’).

For the expressive language sub‐scale, item 41 (names four colours) did not scale at either time point. The following items did not scale at the 30‐month time point but did scale at the 42‐month time point: item 28 (names picture series: five pictures) and item 45 (uses present progressive form). At the 42‐month time point, the following items did not scale: item 32 (poses multiple‐word questions), item 33 (makes a contingent utterance), item 35 (names action picture series: three pictures), item 37 (names action picture series: five pictures) and item 38 (uses plurals).

For the fine motor sub‐scale, the following items did not scale at either time point: item 51 (cuts paper) and item 55 (cuts on a line). The following items did not scale at 30 months but did scale at the 42‐month time point: item 35 (coins in slot), item 36 (connecting blocks: apart), item 40 (imitates stroke series: horizontal), item 41 (imitates stroke series: vertical), item 43 (imitates stroke series: circular), item 46 (builds train of blocks) and item 62 (taps finger). Only one child passed item 62. At 42 months, the following items did not scale: item 38 (block stacking series: six blocks), item 47 (snips paper), item 48 (grasp series: dynamic grasp) and item 49 (tactilely discriminates shapes).

For the gross motor sub‐scale, item 56 (walks forward on path) did not scale at the 30‐month time point but did scale at the 42‐month time point. At the 42‐month time point, the following items did not scale: item 62 (walks on tiptoes 4 steps), item 63 (walks backwards close to path), item 69 (balances on right foot series: 8 s, alone) and item 70 (balances on left foot series: 8 s, alone).

Details of the items considered to lack content and cultural validity are presented in the Supporting Information File 1.

### Convergent validity: associations with age and stunting

The mean score for each Bayley sub‐scale and the total Bayley Scale was significantly lower in 30‐month‐old children compared with 42‐month‐olds (Table 3).

**Table 3:** Mean difference in Bayley Scales scores by age

Bayley sub‐scale	Mean difference in scores from 42 to 30 months (95% confidence interval)	Paired *t*‐test	*P‐*value
Cognitive	5.78 (5.39, 6.17)	29.17	<0.001
Language	13.31 (12.67, 13.96)	40.29	<0.001
Motor	13.92 (13.33, 14.52)	46.05	<0.001
Total scale score	33.02 (31.68, 34.35)	48.69	<0.001

Child stunting was associated with a significantly lower overall score on the total Bayley scale, as well as lower scores on the cognitive sub‐scales at both time points. The language and motor sub‐scale scores were significantly lower in stunted children but only in the older age group (Table 4).

**Table 4:** Mean difference in Bayley scale scores by child stunting status

Bayley sub‐scale	Mean difference in Bayley score (stunted vs. not stunted)	Independent samples *t*‐test	*P*‐value
30 months			
Cognitive	−1.10 (−1.85, −0.35)	−2.88	0.004
Language	−0.91 (−2.09, 0.28)	−1.50	0.134
Motor	−0.58 (−1.66, 0.50)	−1.05	0.294
Total scale score	−2.58 (−5.10, −0.07)	−2.02	0.044
42 months			
Cognitive	−0.83 (−1.42, −0.23)	−2.72	0.007
Language	−1.34 (−2.16, −0.53)	−3.25	0.001
Motor	−1.70 (−2.72, −0.69)	−3.30	0.001
Total scale score	−3.87 (−5.81, −1.94)	−3.93	<0.001

## Discussion

In this study, from a predominantly rural area of Ethiopia, an adapted version of a standardized, observer‐administered and international measure of early child development, the Bayley Scales of Infant Development (version 3), was found to be reliably administered by high school graduates and to have convergent and construct validity for children aged 30 and 42 months. A limitation of our study is that children were only assessed at 30 and 42 months, thus precluding a full evaluation of the Bayley Scale across all age ranges.

Although not all Bayley Scale items scaled onto unidimensional, hierarchical sub‐scales, the scores on the Bayley sub‐scales did distinguish between children of differing ages (30 vs. 42 months). There was also a significant difference in scores between children who were stunted vs. not stunted, with respect to cognitive development (30 and 42 months), and for language and motor development (at 42 months), although the difference was not significant for language and motor development at 30 months. Undernutrition has been more robustly associated with cognitive development in the literature (Martins *et al*. 2011), which may explain this apparent discrepancy. Thus, there was evidence of convergent validity of the adapted Bayley Scales with both age and undernutrition in this population. Furthermore, with the exception of the gross motor sub‐scale, even when the non‐scaling items were excluded, the resulting sub‐scales had good internal consistency and formed adequate‐strong hierarchical scales. The performance of the gross motor scale, particularly in the older age group of children (42 months) was compromised by the need to exclude all items involving stairs. For an inaccessible rural setting where the use of standardized steps is not feasible, alternative indicators of gross motor development are needed.

Some items on the Bayley scales were clearly problematic in this rural Ethiopian setting, particularly those involving the use of scissors (items 47, 51, 55, 64 and 65 of the fine motor sub‐scale), the names for colours (receptive language sub‐scale item 35 and item 68 on the cognitive sub‐scale), use of plurals and understanding pronouns (his or her). Scissors are very rarely present in rural households in Ethiopia meaning that children would lack familiarity with their correct use. A tendency for Ethiopian parents not to linguistically differentiate a wide range of colours was also noted by the Ethiopian co‐investigators, which may serve to explain the low performance on items requiring use of the names of colours. In the Amharic language, although plurals are indicated by a suffix of ‘och’, there use is not idiomatic. His or her pronouns are clearly distinguished in Amharic, and so, it is not apparent why this item might have caused difficulty. In rural Malawi, items relating to knowledge of colours and use of plurals were considered to lack face validity by key informants participating in formative work to develop a locally valid measure of early child development (Gladstone *et al*. 2008).

Removing the time limit for completion of items did not appear to affect the scalability of the items in the cognitive and fine motor sub‐scales but may have affected the scalability of the two gross motor items (located at the difficult end of the scale for the age group) by making them easier to pass. The modifications made to the picture book and stimulus book did not appear to affect the scalability of the majority of items. Few 42‐month‐old children in this sample passed items with the highest level of difficulty in the cognitive sub‐scale, in particular, and to a lesser extent the language sub‐scales. This is to be expected given the high level of stunting in the study population, which is known to be associated with developmental delay particularly in the domain of cognition (Grantham‐McGregor and Baker‐Henningham 2005).

It has been argued that evaluation of early child development in LMICs should make use of internationally standardized measures so as to allow comparison across countries (Reyes *et al*. 2010). For such an approach to be valid, both the construct of early child development and the psychometric properties of the instruments used to measure early child development need to be invariant across countries. As far as we could discern, our study is the first attempt to evaluate the construct and convergent validity of an observational, gold standard measures of child development in a low‐income country setting. Studies from rural, community settings in Kenya (Abubakar *et al*. 2008) and Bangladesh (Hamadani *et al*. 2010) have demonstrated the validity of maternal‐report measures of early childhood development adapted from instruments developed in the West. However, maternal report may be less valid in settings where the background level of awareness about child development is low (Abubakar *et al*. 2008) and may be associated with negative cognitive bias if the mother is depressed. Exploratory qualitative in‐depth interviews with mothers whose child had been administered the Bayley Scales in our study found that mothers wished to portray their child in a positive light. Attempts to develop new measures of early child development, specific to a sociocultural setting, have been made. In rural Malawi, extensive formative work was carried out to understand how child development is seen in that setting and to generate new items to tap into socioculturally valued domains of development (Gladstone *et al*. 2008, 2010a). These new items were combined with those items from international measures of child development that were considered to have face and content validity. Psychometric testing of the resulting child development measure found that 86% of the items drawn from international measures were retained in the scale, and that many of the new, contextually specific items had low reliability and were gender‐specific. That said, the new culture‐specific items comprised more than half of the scale measuring personal‐social domain of development. Therefore, the adequacy of international scales to measure child development may depend to some extent on the domain of development under consideration.

Important drawbacks of using international observational measures of child development include the expense and difficulty of transporting and replacing the equipment, the time‐consuming nature of the assessment and the need for highly skilled testers. Our study has shown that high school graduates can administer the Bayley Scales as reliably as nurses after a relatively short period of training combined with ongoing careful supervision. We found that it was feasible to carry out Bayley Scale assessments in the home setting and that the Scale scores were meaningful. While briefer, more culturally nuanced measures of child development meet a particular need and may be particularly necessary for exploring personal‐social development, there remains a place for standardized, international observational measures. Our study indicates that, with careful adaptation, many of the items from such measures are reliable and valid in a rural Ethiopian setting, thus allowing for comparability across settings.

**Key messages**

- Observational, standardized measures of early child development are the gold standard but may not be valid or feasible for a rural African setting
- An international, observational measure of child development was adapted for rural Ethiopia
- High school graduates were able to administer the measure as reliably as clinical nurses
- Problem items included those examining colour recognition, use of scissors and plurals
- The adapted measure of early child development was shown to be valid and feasible

## Conflict of interest

No conflicts of interest are reported by the authors.

## Supporting information


**Supplementary Table S1:** Item frequencies, content and construct validity for cognitive sub‐scale at 2.5 and 3.5 years of age (X indicates item lacked validity).
**Supplementary Table S2:** Item frequencies, content and construct validity for receptive language sub‐scale at 2.5 and 3.5 years of age (X indicates item lacked validity).
**Supplementary Table S3:** Item frequencies, content and construct validity for expressive language sub‐scale at 2.5 and 3.5 years of age (X indicates item lacked validity).
**Supplementary Table S4:** Item frequencies, content and construct validity for fine motor sub‐scale at 2.5 and 3.5 years of age (X indicates item lacked validity).
**Supplementary Table S5:** Item frequencies, content and construct validity for gross motor sub‐scale at 2.5 and 3.5 years of age (shaded items not administered and X indicates item lacked validity).
