# Supplementary material for: Adapting the Bayley Scales of infant and toddler development in Ethiopia: evaluation of reliability and validity
Source: Child Care Health Dev. 2016 Jul 6;42(5):699–708. doi: 10.1111/cch.12371 (PMC4979664; doi:10.1111/cch.12371)
Supplement: Supplementary file 1 — Supplementary Table S1: Item frequencies, content and construct validity for cognitive sub‐scale at 2.5 and 3.5 years of age (X indicates item lacked validity). Supplementary Table S2: Item frequencies, content and construct validity for receptive language sub‐scale at 2.5 and 3.5 years of age (X indicates item lacked validity). Supplementary Table S3: Item frequencies, content and construct validity for expressive language sub‐scale at 2.5 and 3.5 years of age (X indicates item lacked validity). Supplementary Table S4: Item frequencies, content and construct validity for fine motor sub‐scale at 2.5 and 3.5 years of age (X indicates item lacked validity). Supplementary Table S5: Item frequencies, content and construct validity for gross motor sub‐scale at 2.5 and 3.5 years of age (shaded items not administered and X indicates item lacked validity). [file CCH-42-699-s001.docx]

Supplementary table 1: Item frequencies, content and construct validity for cognitive sub-scale at 2.5 and 3.5 years of age (X indicates item lacked validity)

| **Item** | **Brief descriptor of item** | **% passing item** | | **Content validity^†^** | | **Construct validity^±^** | |
| --- | --- | --- | --- | --- | --- | --- | --- |
|  |  | **2.5 year time-point (n=440)** | **3.5 year time-point (n=456)** | **2.5 year time-point (n=440)** | **3.5 year time-point (n=456)** | **2.5 year time-point (n=440)** | **3.5 year time-point (n=456)** |
|  | *Start point for children aged 28 months 16 days – 32 months 30 days* |  |  |  |  |  |  |
| 60 | Rotated pink board | 93.9 | - |  |  |  |  |
| 61 | Object assembly: ball^*^ | 96.6 | - |  |  |  |  |
| 62 | Completes pegboard: 25 seconds^*^ | 99.6 | - |  |  |  |  |
| 63 | Object assembly (ice cream cone)^*^ | 90.9 | - |  |  |  |  |
| 64 | Matches pictures | 47.3 | - |  |  | X |  |
| 65 | Representational play | 70.9 | - |  |  | X |  |
| 66 | Blue board series: completes (75 seconds)^*^ | 75.5 | - |  |  |  |  |
|  | *Start point for children aged 39 months 0 days –*  *42 months 15 days* |  |  |  |  |  |  |
| 67 | Imitates a 2-step action | 73.2 | 93.6 |  |  | X |  |
| 68 | Matches 3 colours | 2.7 | 6.8 | X | X | X | X |
| 69 | Imaginary play | 58.9 | 86.8 |  |  | X |  |
| 70 | Understands concept of one^*^ | 36.8 | 77.0 |  |  |  |  |
| 71 | Multischeme combination play | 51.8 | 83.8 |  |  |  |  |
| 72 | Concept grouping: colour | 17.1 | 54.4 |  |  |  |  |
| 73 | Concept grouping: size | 15.2 | 78.1 |  |  |  |  |
| 74 | Compares masses | 18.9 | 75.7 |  |  |  |  |
| 75 | Matches size | 6.1 | 43.4 |  |  |  |  |
| 76 | Discriminates pictures | 10.5 | 66.5 |  |  |  | X |
| 77 | Simple pattern | 5.9 | 46.9 |  |  |  |  |
| 78 | Sorts pegs by colour | 2.1 | 16.5 |  |  |  |  |
| 79 | Counts (one-to-one correspondence) | 3.2 | 10.3 |  |  |  |  |
| 80 | Discriminates sizes | 0.2 | 4.0 |  |  |  |  |
| 81 | Identifies 3 complete pictures | 0 | 0.2 |  |  |  | X |
| 82 | Object assembly (dog)^*^ | 0 | 0 |  |  |  | X |
| 83 | Discriminates patterns | 0 | 0.2 |  |  |  |  |
| 84 | Spatial memory | 0 | 0 |  |  |  | X |
| 85 | Counts (cardinality) | 0 | 0.2 |  |  |  |  |
| 86 | Number constancy | 0 | 0 |  |  |  | X |
| 87 | Laces card | 0 | 0.2 |  |  |  |  |
| 88 | Classifies objects | 0 | 0 |  |  |  | X |
| 89 | Understands concept of more | 0 | 0.2 |  |  |  |  |
| 90 | Repeats number sequences | 0 | 0 |  |  |  |  |
| 91 | Completes patterns | 0 | 0 |  |  |  |  |

^*^time limit was not applied, ^†^Content validity indicated by frequency of passing relative to neighbouring items, ^±^Construct validity indicated by scaling on hierarchical Mokken scale

Supplementary table 2: Item frequencies, content and construct validity for receptive language sub-scale at 2.5 and 3.5 years of age (X indicates item lacked validity)

| **Item** | **Brief descriptor of item** | **% passing item** | | **Content validity^†^** | | **Construct validity^±^** | |
| --- | --- | --- | --- | --- | --- | --- | --- |
|  |  | **2.5 year time-point (n=440)** | **3.5 year time-point (n=456)** | **2.5 year time-point (n=440)** | **3.5 year time-point (n=456)** | **2.5 year time-point (n=440)** | **3.5 year time-point (n=456)** |
|  | *Start point for children aged 28 months 16 days – 32 months 30 days* |  |  |  |  |  |  |
| 22 | Identifies 3 clothing items | 98.9 | - |  |  | X |  |
| 23 | Identifies action picture series: 1 correct | 95.7 | - |  |  |  |  |
| 24 | Identifies 5 parts of the body | 98.2 | - |  |  |  |  |
| 25 | Follows 2-part directions | 82.1 | - |  |  |  |  |
| 26 | Identifies action picture series: 3 correct | 55.9 | - |  |  |  |  |
| 27 | Understands use of objects | 25.5 | - |  |  |  |  |
|  | *Start point for children aged 39 months 0 days –*  *42 months 15 days* |  |  |  |  |  |  |
| 28 | Understands part/whole relationships | 29.6 | 96.5 |  |  |  |  |
| 29 | Identifies action picture series: 5 correct | 20.2 | 94.7 |  |  |  |  |
| 30 | Understands pronouns (Him, Me, My, You, Your) | 37.5 | 96.5 |  |  |  |  |
| 31 | Understands labels for sizes | 13.0 | 87.3 |  |  |  |  |
| 32 | Understands prepositions series: 2 correct | 43.2 | 97.4 |  |  |  |  |
| 33 | Understands possessives | 0.9 | 29.2 |  |  |  | X |
| 34 | Understands verb + ing | 0.7 | 33.3 |  |  |  | X |
| 35 | Identifies colours | 0 | 0.2 |  | X | X | X |
| 36 | Understands labels of one | 0.7 | 41.9 |  |  |  |  |
| 37 | Understands pronouns (They, She, He) | 0.2 | 33.3 |  |  |  |  |
| 38 | Understands pronouns (His, Her) | 0 | 2.0 |  | X | X |  |
| 39 | Understands plurals | 0.2 | 18.0 |  |  |  |  |
| 40 | Understands more | 0 | 19.5 |  |  | X |  |
| 41 | Understands most | 0.2 | 7.9 |  |  |  |  |
| 42 | Understands prepositions series: 4 correct | 0 | 33.1 |  |  |  |  |
| 43 | Understands negatives in sentences | 0 | 4.4 |  |  |  |  |
| 44 | Understands past tense | 0 | 1.5 |  |  |  |  |
| 45 | Understands labels for mass | 0 | 3.7 |  |  |  |  |
| 46 | Understands least | 0 | 3.5 |  |  |  |  |
| 47 | Understands less | - | 3.1 |  |  |  |  |
| 48 | Understands descriptive labels | - | 0.4 |  |  |  |  |

^†^Content validity indicated by frequency of passing relative to neighbouring items, ^±^Construct validity indicated by scaling on hierarchical Mokken scale

Supplementary table 3: Item frequencies, content and construct validity for expressive language sub-scale at 2.5 and 3.5 years of age (X indicates item lacked validity)

| **Item** | **Brief descriptor of item** | **% passing item** | | **Content validity^†^** | | **Construct validity^±^** | |
| --- | --- | --- | --- | --- | --- | --- | --- |
|  |  | **2.5 year time-point (n=440)** | **3.5 year time-point (n=456)** | **2.5 year time-point (n=440)** | **3.5 year time-point (n=456)** | **2.5 year time-point (n=440)** | **3.5 year time-point (n=456)** |
|  | *Start point for children aged 28 months 16 days – 32 months 30 days* |  |  |  |  |  |  |
| 23 | Uses word appropriately series: 8 words | 88.9 | - |  |  |  |  |
| 24 | Answers yes or no verbally in response to questions | 94.8 | - |  |  |  |  |
| 25 | Imitates as two-word utterance | 87.7 | - |  |  |  |  |
| 26 | Uses a two-word utterance | 74.6 | - |  |  |  |  |
| 27 | Names object series: 3 objects | 77.7 | - |  |  |  |  |
| 28 | Names picture series: 5 pictures | 43.9 | - |  |  | X |  |
| 29 | Uses multiple word utterances | 62.3 | - |  |  |  |  |
|  | *Start point for children aged 39 months 0 days –*  *42 months 15 days* |  |  |  |  |  |  |
| 30 | Uses pronouns | 46.1 | 97.6 |  |  |  |  |
| 31 | Names action picture series: 1 picture | 39.6 | 88.6 |  |  |  |  |
| 32 | Poses multiple-word questions | 47.5 | 93.4 |  |  |  | X |
| 33 | Makes a contingent utterance | 36.8 | 64.0 |  |  |  | X |
| 34 | Uses verb +ing | 5.0 | 54.4 |  |  |  |  |
| 35 | Names action picture series: 3 pictures | 10.2 | 47.2 |  |  |  | X |
| 36 | Uses different word combinations | 15.5 | 61.0 |  |  |  |  |
| 37 | Names action picture series: 5 pictures | 1.1 | 26.3 |  |  |  | X |
| 38 | Uses plurals | 0.7 | 2.6 |  | X |  | X |
| 39 | Answers what and where questions | 0.9 | 33.3 |  |  |  |  |
| 40 | Uses possessives | 1.1 | 21.5 |  |  |  |  |
| 41 | Names 4 colours | 0 | 0.2 |  | X | X | X |
| 42 | Answers questions logically (related to functions) | 0.2 | 30.9 |  |  |  |  |
| 43 | Tells how an object is used | 0.2 | 2.2 |  |  |  |  |
| 44 | Uses prepositions | 0.5 | 9.7 |  |  |  |  |
| 45 | Uses present progressive form | 0 | 0.9 |  |  | X |  |
| 46 | Describes picture series: uses 4-5 word sentences | 0.2 | 0.7 |  |  |  |  |
| 47 | Describes picture series: uses past tense | 0 | 0.4 |  |  |  |  |
| 48 | Describes picture series: uses future tense | 0 | 0.4 |  |  |  |  |

^†^Content validity indicated by frequency of passing relative to neighbouring items, ^±^Construct validity indicated by scaling on hierarchical Mokken scale

Supplementary table 4: Item frequencies, content and construct validity for fine motor sub-scale at 2.5 and 3.5 years of age (X indicates item lacked validity)

| **Item** | **Brief descriptor of item** | **% passing item** | | **Content validity^†^** | | **Construct validity^±^** | |
| --- | --- | --- | --- | --- | --- | --- | --- |
|  |  | **2.5 year time-point (n=440)** | **3.5 year time-point (n=456)** | **2.5 year time-point (n=440)** | **2.5 year time-point (n=440)** | **3.5 year time-point (n=456)** | **2.5 year time-point (n=440)** |
|  | *Start point for children aged 28 months 16 days – 32 months 30 days* |  |  |  |  |  |  |
| 35 | Coins in slot | 99.6 | - |  |  | X |  |
| 36 | Connecting blocks: apart | 96.6 | - |  |  | X |  |
| 37 | Grasp series: intermediate (tripod) grasp | 92.1 | - |  |  |  |  |
| 38 | Block stacking series: 6 blocks | 95.9 | - |  |  |  | X |
| 39 | Uses hand to hold paper in place | 60.0 | - |  |  |  |  |
| 40 | Imitates stroke series: horizontal | 51.8 | - |  |  | X |  |
| 41 | Imitates stroke series: vertical | 39.3 | - |  |  | X |  |
| 42 | Connecting blocks: together | 31.6 | - |  |  |  |  |
|  | *Start point for children aged 39 months 0 days –*  *42 months 15 days* |  |  |  |  |  |  |
| 43 | Imitates strokes series: circular | 51.8 | 97.2 |  |  | X |  |
| 44 | Builds train of blocks | 51.4 | 97.8 |  |  |  |  |
| 45 | Strings 3 blocks | 65.0 | 99.6 |  |  |  |  |
| 46 | Imitates hand movements | 27.7 | 93.6 |  |  | X |  |
| 47 | Snips paper | 0.2 | 2.6 | X | X |  | X |
| 48 | Grasp series: dynamic grasp | 10.5 | 57.0 |  |  |  | X |
| 49 | Tactilely discriminates shapes | 4.8 | 51.3 |  |  |  | X |
| 50 | Builds wall | 14.6 | 77.0 |  |  |  |  |
| 51 | Cuts paper | 0.5 | 1.1 | X | X | X | X |
| 52 | Builds bridge | 11.6 | 73.9 |  |  |  |  |
| 53 | Imitates plus sign | 1.6 | 34.4 |  |  |  |  |
| 54 | Block stacking series: 8 blocks | 13.0 | 79.4 |  |  |  |  |
| 55 | Cuts on line | 0 | 0.4 | X | X | X | X |
| 56 | Builds T | 3.4 | 34.7 |  |  |  |  |
| 57 | Buttons: 1 button | 0.5 | 14.5 |  |  |  |  |
| 58 | Builds steps | 2.5 | 12.9 |  |  |  |  |
| 59 | Traces designs | 0.2 | 3.5 |  |  |  |  |
| 60 | Imitates square | 0.2 | 1.3 |  |  |  |  |
| 61 | Copies plus sign | 0.7 | 2.2 |  |  |  |  |
| 62 | Taps finger* | 0 | 2.9 |  |  | - |  |
| 63 | Places 20 pellets in bottle* | 0 | 4.0 |  |  | - |  |
| 64 | Cuts circle* | 0 | 0 |  |  | - | - |
| 65 | Cuts square* | 0 | 0 |  |  | - | - |
| 66 | Copies square | 0 | 0 |  |  | - | - |

*time limit was not applied; ^†^Content validity indicated by frequency of passing relative to neighbouring items, ^±^Construct validity indicated by scaling on hierarchical Mokken scale

Supplementary table 5: Item frequencies, content and construct validity for gross motor sub-scale at 2.5 and 3.5 years of age (shaded items not administered and X indicates item lacked validity)

| **Item** | **Brief descriptor of item** | **% passing item** | | **Content validity^†^** | | **Construct validity^±^** | |
| --- | --- | --- | --- | --- | --- | --- | --- |
|  |  | **2.5 year time-point (n=440)** | **3.5 year time-point (n=456)** | **2.5 year time-point (n=440)** | **2.5 year time-point (n=440)** | **3.5 year time-point (n=456)** | **2.5 year time-point (n=440)** |
|  | *Start point for children aged 28 months 16 days – 32 months 30 days* |  |  |  |  |  |  |
| 51 | Balances on right foot series: with support | 97.7 | - |  |  |  |  |
| 52 | Balances on left foot series: with support | 96.1 | - |  |  |  |  |
| 53 | Walks sideways without support | 95.5 | - |  |  |  |  |
| 54 | Jumps from bottom step | - | - |  |  |  |  |
| 55 | Kicks ball | 93.2 | - |  |  |  |  |
| 56 | Walks forward on path | 78.9 | - |  |  | X |  |
|  | *Start point for children aged 39 months 0 days –*  *42 months 15 days* |  | - |  |  |  |  |
| 57 | Walks up stairs series: both feet on each step, alone | - | - |  |  |  |  |
| 58 | Walks down stairs series: both feet on each step, alone | - | - |  |  |  |  |
| 59 | Jumps forward series: 4 inches | 21.1 | 90.4 |  |  |  |  |
| 60 | Balances on right foot series: 2 seconds, alone | 59.6 | 99.6 |  |  |  |  |
| 61 | Balances on left foot series: 2 seconds, alone | 51.1 | 99.1 |  |  |  |  |
| 62 | Walks on tiptoes 4 steps | 9.3 | 48.7 |  |  |  | X |
| 63 | Walks backwards close to path | 23.0 | 75.7 |  |  |  | X |
| 64 | Walks up stairs series: alternating feet, alone | - | - |  |  |  |  |
| 65 | Imitates postures | 20.0 | 83.3 |  |  |  |  |
| 66 | Stops from a full run | 13.6 | 49.1 |  |  |  |  |
| 67 | Walks down stairs series: alternating feet, alone | - | - |  |  |  |  |
| 68 | Hops 5 feet | 2.5 | 25.2 |  |  |  |  |
| 69 | Balances on right foot series: 8 seconds, alone* | 10.9 | 35.5 |  |  |  | X |
| 70 | Balances on left foot series: 8 seconds, alone* | 1.8 | 14.0 |  |  |  | X |
| 71 | Walks heel to toe | 6.8 | 26.8 |  |  |  |  |
| 72 | Jumps forward series: 24 inches | 3.4 | 23.7 |  |  |  |  |

*time limit was not applied; ^†^Content validity indicated by frequency of passing relative to neighbouring items, ^±^Construct validity indicated by scaling on hierarchical Mokken scale
